# i-RheoFT: Fourier transforming sampled functions without artefacts

**DOI:** 10.1038/s41598-021-02922-8

**Published:** 2021-12-15

**Authors:** Matthew G. Smith, Graham M. Gibson, Manlio Tassieri

**Affiliations:** 1 Division of Biomedical Engineering, James Watt School of Engineering, University of Glasgow, Glasgow, G12 8LT UK; 2 SUPA, School of Physics and Astronomy, University of Glasgow, Glasgow, G12 8QQ UK

**Keywords:** Engineering, Mathematics and computing, Physics

## Abstract

In this article we present a new open-access code named “i-RheoFT” that implements the analytical method first introduced in [PRE, 80, 012501 (2009)] and then enhanced in [New J Phys 14, 115032 (2012)], which allows to evaluate the Fourier transform of any generic time-dependent function that vanishes for negative times, sampled at a finite set of data points that extend over a finite range, and *need not* be equally spaced. I-RheoFT has been employed here to investigate three important experimental factors: (i) the ‘density of initial experimental points’ describing the sampled function, (ii) the interpolation function used to perform the “*virtual oversampling*” procedure introduced in [New J Phys 14, 115032 (2012)], and (iii) the detrimental effect of noises on the expected outcomes. We demonstrate that, at relatively high signal-to-noise ratios and density of initial experimental points, all three built-in MATLAB interpolation functions employed in this work (i.e., Spline, Makima and PCHIP) perform well in recovering the information embedded within the original sampled function; with the Spline function performing best. Whereas, by reducing *either* the number of initial data points *or* the signal-to-noise ratio, there exists a threshold below which all three functions perform poorly; with the worst performance given by the Spline function in both the cases and the least worst by the PCHIP function at low density of initial data points and by the Makima function at relatively low signal-to-noise ratios. We envisage that i-RheoFT will be of particular interest and use to all those studies where sampled or time-averaged functions, often defined by a discrete set of data points within a finite time-window, are exploited to gain new insights on the systems’ dynamics.

## Introduction

In the field of soft-matter physics, it has been shown that at thermal equilibrium the motion and the interactions between the materials’ building blocks govern the linear mechanical properties of matter^1–3^. These can be educed via a statistical mechanics (SM) analysis of the thermally driven motion of their constituents (e.g., molecules); whose dynamics can be measured either directly (e.g., neutron spin echo^4,5^) or implicitly by means of (nano/micro) tracers embedded into the samples^6^. Interestingly, the majority of these experimental methods return a measure of the materials’ dynamics in an analytical form of a time-dependent exponential decay function, which in the simplest cases assumes the shape of a ‘single’ exponential decay $exp(-t/\tau _c)$, with $\tau _c$ being the characteristic relaxation time of the process under investigation (e.g., the diffusion of monodisperse molecules/tracers in Newtonian fluids^7^ or bond percolation of transient gels^8^).

In general, for more complex systems than those just mentioned, a SM analysis of materials’ thermal fluctuations may return more convoluted functions, such as a ‘stretched’ exponential $exp(-t/\tau _c)^{\beta }$, with $\beta <1$. This is indeed a common outcome of both dynamic light scattering (DLS)^9^ and diffusing wave spectroscopy (DWS)^10^ measurements; e.g., when employed in the study of the dynamics of semi-flexible biopolymer solutions^11^, for which the high-frequency mechanical properties are expected to show a frequency ($\omega$) dependency of the linear viscoelastic (LVE) moduli proportional to $\omega ^{\beta }$, with $\beta = 0.75$^12^. By increasing systems’ complexity, such as in the field of polymer physics, a SM analysis of the shear component of the stress tensor allows to evaluate the time-dependent materials’ shear relaxation modulus *G*(*t*)^13^. This is often a multi-modal decay function (i.e. characterised by multiple relaxation times) that embodies, without disclosing at once, the full frequency spectrum of the materials’ LVE properties. These are instead fully revealed by the frequency-dependent materials’ complex shear modulus $G^*(\omega )$, which is a complex number whose real and imaginary parts provide quantitative information on the elastic and viscous nature of the material, respectively^14^. Notably, these two moduli are in principle simply related to each other by means of the Fourier transform of the time derivative of *G*(*t*), whose computation given a discrete set of data is at the heart of this article. A similar issue is encountered in the field of microrheology^15^, where in the particular case of measurements performed with optical tweezers, a SM analysis of the trajectory of an optically trapped particle suspended into a complex fluid may return the particle normalised position autocorrelation function $A(\tau )$, or equivalently its normalised mean square displacement $\Pi (\tau )=1-A(\tau )$ (where $\tau$ is the lag-time or time interval), whose temporal form is a generic decay (or growth) function governed by the frequency-dependent linear viscoelastic properties of the suspending fluid^16^.

Interestingly, a common feature for all the above mentioned time-dependent functions is that they are evaluated for a discrete number of timestamps, within a finite observation time window. Yet, one of the aims of most of the studies where they are employed is often to evaluate the ‘continuous’ frequency spectrum of the system, over the widest range of experimentally accessible frequencies. Thus the need of an effective Fourier transform algorithm to translate the information embedded into a generic time-dependent sampled function into those equipollent, but more explicit, in the frequency-domain. This is a non-trivial task^17^, and has driven scientists to overcome such an issue by fitting the experimental data by means of a generalized Maxwell model (i.e., a finite sum of weighted single exponentials, each identifying a characteristic relaxation time of the system), which has a straightforward Fourier transform, but may potentially interpret the data^18^. Remarkably, an effective solution to this issue has been presented by Evans et al.^17^ and its efficacy has been augmented by Tassieri et al.^19^ by means of a “*virtual oversampling*” procedure that involves first a numerical interpolation between experimental data points by using a standard non-overshooting cubic spline function, and then generating a new, over-sampled data set, by sampling the interpolating function at a number of equally spaced points on a logarithmic time scale. The effectiveness of this method has been validated for a variety of applications within the fields of rheology and microrheology^17,19^; however, its general validity has been not fully exploited yet.

In this work, we have implemented the analytical method developed by Evans & Tassieri into a open-access MATLAB code named “i-RheoFT” (allowing its use to a broad audience, see SI) and investigated its accuracy as function of three important experimental factors that are often overlooked in many applications: (i) the density of initial experimental points (DIP) describing a generic time-dependent function (i.e., the sampled function), (ii) the interpolation function used to implement the virtual oversampling procedure, and (iii) the destructive effects on the expected outcomes due to the presence of (white) noise.

As we shall demonstrate, the relative value of the first parameter plays a crucial role in the quality of the outputs of all those experimental methods (such as DLS, DWS and NSE) where data are acquired at high frequencies (e.g., at 10^7^ Hz or at 10^9^ Hz in the case of DLS and NSE measurements, respectively) and stored in the form of time-averaged functions, which are often evaluated on-the-fly by means of fast correlators. These correlation functions are commonly evaluated only for a relatively ‘small’ number of lag-times within a finite time window, often spanning several decades (e.g., from 10^−7^ sec to 10^2^ sec in the case of DLS measurements); thus avoiding the risk of clogging the machines’ internal random-access memory (RAM) after a few seconds of measurement duration. The investigation of the second point has been driven by the fact that a few research groups have implemented the oversampling procedure by using different interpolation functions^20,21^ than the one employed in the original work^19^. Therefore, here we have compared the effectiveness of the following three interpolation functions already built-in MATLAB: a cubic spline data interpolation (Spline)^22^ (as the one used in^19^), a modified Akima piecewise cubic Hermite interpolation (Makima)^23^ and Piecewise Cubic Hermite Interpolating Polynomial (PCHIP)^24^. Notably, we can anticipate that, at relatively high DIP values and signal-to-noise ratios (SNR), all three of the above mentioned interpolation methods recover the information embedded into the sampled function to a high degree of fidelity, allowing the evaluation of its Fourier transform without a significant loss of information. Whereas, at relatively low values of *either* of DIP *or* SNR, the same is not true and a clear discrimination between their efficacy is achieved in both the time- and the frequency-domains.

## Theoretical background

### Fourier transform of raw data

In the digital era, continuous data storing does not exist and signals are stored at a finite acquisition rate (AR), whatever fast this process could be. Therefore, measurements are represented by a finite sequence of points, often equally spaced in time and rarely acquired at time intervals equally spaced on a logarithmic scale. Nonetheless, a logarithmic timestamp is often used in post-acquisition storing procedures, such as those employed in the study of fast quasi-stochastic phenomena, for which (i) a high AR is necessary and (ii) prolonged measurements are mandatory because of statistical principles. However, these two requirements would place a high demand on the RAM capacity of any machine. Thus, the common use of correlators to evaluate on-the-fly a correlation function of the acquired signal for a finite set of lag-times often logarithmically spaced within a defined time window that spans several decades; yet, without a need of storing the raw data.

Interestingly, the discrete nature of measurements has revealed to be a hurdle to overcome in many applications, especially for those where a Fourier transform is involved; simply because the latter is a linear (integral) operator that requires the integrating function to be defined $\forall t \in ]-\infty , +\infty [$, and not just for a finite set of timestamps. This is equally true for all those processes where the integrating function is defined only for positive times (i.e., $\forall t \in [0^+, +\infty [$) and it is (or assumed to be) identically equal to zero $\forall t \in ]-\infty , 0^-]$ because of causality; such as in the studies of materials’ relaxation processes after they have been subjected to either a finite deformation or a constant stress (creep), both applied within a small time interval ($\epsilon$)^14,17^. In this regard, an analytical procedure for the evaluation of the Fourier transform of any generic function sampled over a finite time window has been proposed by Evans et al.^17^ to convert creep compliance *J*(*t*) (defined as the ratio between the material strain $\gamma (t)$ and the applied constant stress) into $G^*(\omega )$ directly, without the use of Laplace transforms or fitting functions. This method is based on the interpolation of the finite data set by means of a piecewise-linear function. In particular, the general validity of the proposed procedure makes it equally applicable to find the Fourier transform ${\hat{g}}(\omega )$ of any time-dependent function *g*(*t*) that vanishes for negative *t*, sampled at a finite set of data points $(t_k,g_k)$, where $k=1\ldots N$, which extend over a finite range, and *need not* be equally spaced^17^:

1 $$\begin{aligned}&-\omega ^2 {\hat{g}}\left( \omega \right) = i\omega g(0) + \left( 1-e^{-i\omega t_1}\right) \frac{\left( g_1-g(0)\right) }{t_1} +\nonumber \\&\quad + {\dot{g}}_\infty e^{-i\omega t_N} + \sum _{k=2}^N \left( \frac{g_k-g_{k-1}}{t_k-t_{k-1}} \right) \left( e^{-i\omega t_{k-1}}-e^{-i\omega t_k} \right) \end{aligned}$$

where ${\dot{g}}_\infty$ is the gradient of *g*(*t*) extrapolated to infinite time and *g*(0) is the value of *g*(*t*) extrapolated to $t=0$ from above.

This method has been improved by Tassieri et al.^19^ while analysing microrheology measurements performed with optical tweezers^15^. The authors found that a substantial reduction in the size of the high-frequency artefacts, from which some high-frequency noise tends to spill over into the top of the experimental frequency range, can be achieved by an *over-sampling* technique. The technique involves first numerically interpolating between data points using a standard non-overshooting cubic spline, and then generating a new, over-sampled data set, by sampling the interpolating function at a number of equally-spaced points on either a logarithmic or a linear time-scale. We remind that, over-sampling is a common procedure in signal processing and it consists of sampling a signal with a sampling frequency $f_{s}$ much higher than the Nyquist rate 2*B*, where *B* is the highest frequency contained in the original signal. A signal is said to be oversampled by a factor of $\beta \equiv f_{s} / (2B)$^25^.

### Interpolation functions

In this work we have employed three built-in interpolation functions listed in MATLAB: (i) Makima, (ii) Spline and (iii) Piecewise Cubic Hermite Interpolating Polynomial (PCHIP). These are fully described in Refs.^22–24^ and here they are briefly summarised for convenience of the reader.

*The Makima algorithm* for one-dimensional interpolation, also described in Ref.s^26,27^, is a modification to the Akima algorithm that performs cubic interpolation to produce piecewise polynomials with continuous first-order derivatives. The algorithm preserves the slope and avoids undulations in flat regions. A flat region occurs whenever there are three or more consecutive collinear points, which the algorithm connects with a straight line. When two flat regions with different slopes meet, the modification made to the original Akima algorithm gives more weight to the side where the slope is closer to zero. This modification gives priority to the side that is closer to horizontal, which is more intuitive and avoids overshoot. Notice that, the original Akima algorithm gives equal weights to the points on both sides, thus evenly dividing the undulation.

*The Spline algorithm*, performs cubic interpolation to produce piecewise polynomials with continuous second-order derivatives. The result is comparable to a regular polynomial interpolation, but is less susceptible to heavy oscillation between data points for high degrees. Still, this method can be susceptible to overshoots and oscillations between data points at long times. Interestingly, when this is compared to the Akima algorithm, the latter produces fewer undulations and is better suited to deal with quick changes between flat regions.

*The PCHIP algorithm*, also performs a piecewise cubic polynomial interpolation in much the same way as the Spline function just mentioned. However, they differ in one key area which is that while the Spline functions’ second-order derivative is continuous, the second-order derivative for PCHIP is unlikely to be and therefore the interpolation function is shape preserving for large jumps between data points. Additionally, the non continuous nature of the second-derivative means that PCHIP has no overshoots and much lower oscillation when the data is not smooth when compared to the Spline function.

### Sampled functions and the density of initial points

**Figure 1:**
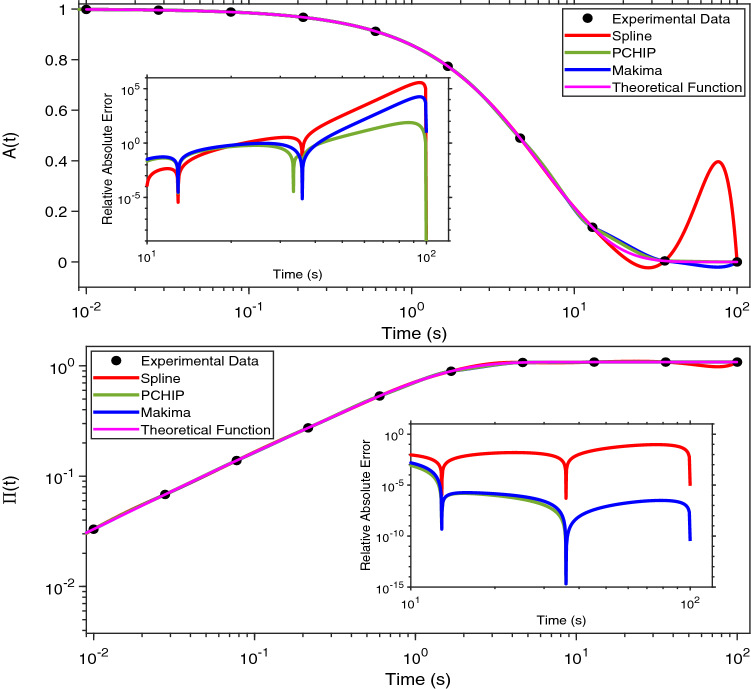
(Top) A single exponential decay function vs. time, representing the relaxation modulus of a single mode Maxwell fluid. (Bottom) A generic function resembling the normalised mean square displacement vs. time of an optically trapped particle suspended into a non-Newtonian fluid. Equations (2) and (3) are represented by a finite number of ‘sampled’ points and a continuous (pink) line. The points are also interpolated by means of three MATLAB built-in interpolation functions: Spline, PCHIP and Makima. The insets show the relative absolute error of each interpolation function with respect of either of Eqs. (2) and (3), as calculated using Eq. (7). The time window of the inset encompasses the final three points of the main graph, where the relative error is at its highest.

In order to quantify the fidelity of the above mentioned interpolation procedures in recovering the original information contained by a sampled function, we have employed them to evaluate the Fourier transform (via Eq. 1) of two functions that are similar to those often seen in optical tweezers experiments^15,16^: (I) a single exponential decay function:

2 $$\begin{aligned} A(t)=exp(-t/\tau _c), \end{aligned}$$

describing the dynamics of a Maxwell fluid characterised by a single relaxation time $\tau _c$, as shown in Fig. 1 (top); and (II) a set of data resembling the mean square displacement of a weakly trapped probe particle suspended into a non-Newtonian fluid (Fig. 1 (bottom)), which have been evaluated by means of the following series:

3 $$\begin{aligned} \Pi (q,t)=\sum ^\infty _{q=1}\frac{1}{q^4}\Big (1-e^{-q^4t}\Big ),~~~t\geqslant 0, \end{aligned}$$

where *q* is the mode number and time is measured in units of the longest relaxation time for $q=1$. It is important to notice that Eq. (3) is a series definitely convergent, and in this work we have used its approximant $\Pi _{11}(q, t) = \sum _{q=1}^{11} (1-e^{-tq^4}) q^{-4}$; which provides a good estimation of the series $\forall t$. This has been corroborated by evaluating the incremental value of the mean relative error of the approximant $\Pi _{11}(q, t)$ when compared to the approximants $\Pi _{100}(q, t)$ and $\Pi _{200}(q, t)$, for $t\in [10^{-2},10^2]$; which results in being as low as $0.0000287\%$.

Interestingly, the Fourier transform of the time derivative of both the functions described by Eqs. (2) and (3) can be calculated analytically and therefore an exact expression of the related complex moduli (i.e., $G_A^*(\omega )$ and $G_\Pi^*(\omega )$, describing the viscoelastic properties of the suspending fluids) can be derived for both of them^15,16,18^:

4 $$\begin{aligned} {G_A^*(\omega )=i\omega {\hat{A}}(\omega ) =\frac{(\omega \tau _c)^2}{1+(\omega \tau _c)^2}+i\frac{\omega \tau _c}{1+(\omega \tau _c)^2}} \end{aligned}$$

and

5 $$\begin{aligned} {G_\Pi^*(\omega )=[i\omega {\hat{\Pi }}(\omega )]^{-1} =\Bigg [\sum ^{11}_{q=1}\frac{(q^4-i\omega )}{(q^8+\omega ^2)}\Bigg ]^{-1}} \end{aligned}$$

where ${\hat{A}}(\omega )$ and ${\hat{\Pi }}(\omega )$ are the Fourier transforms of the functions described by Eqs. (2) and (3), respectively. It follows that, Eqs. (4) and (5) can each act as a reference for a quantitative evaluation of the errors generated during the Fourier transform of a discrete set of data representing either of Eqs. (2) or (3), as function of both the density of initial points (DIP) and the oversampling factor ($\beta$). However, while the latter is a well known parameter in signal processing and would not affect the effectiveness of Eq. (1) for relatively high values of $\beta$^19^ (here it is kept constant to 50), DIP is introduced in this work to inform the scientific community of its relevance when modelling or interpreting a discrete set of data by means of a continuous interpolation function:

6 $$\begin{aligned} DIP = \frac{\log _{10}({N_e})}{\log _{10}(t_N/t_1)} \end{aligned}$$

where $N_e$ is the number of experimental data points within the explored time window, which extends from a lower end equal to $t_1$ to a maximum time equal to $t_N$. Therefore, a function sampled at a few MHz over a time window spanning from $t_1=10^{-7}$s to $t_N=10$s would have DIP$=1$ if $N_e$ were equal to 10^8^. Interestingly, this is not the case for the majority of the experimental techniques mentioned in the introduction (e.g. DLS, DWS, etc.), for which the technological constrain dictated by the finite RAM of the instruments is compensated by the adoption of correlators that *convolve* the high-speed acquired data into a finite set of *averaged* values calculated for a relatively small number of lag-times. Thus, returning a DIP value often much smaller than 1; which, as we shall demonstrate below, may lead to erroneous outcomes, especially at relatively low DIP values.

## Results and discussion

Let us start by considering both Eqs. (2) and (3) sampled at a relatively low acquisition rate, as shown in Fig. 1. Both the functions are represented by 10 experimental points equally spaced on a logarithmic scale (black dots) and a continuous (pink) line, within a time window ranging from 10^−2^ to 10^2^s; which implies a DIP$=1/4$.

The experimental data have been interpolated by using the three MATLAB built-in functions mentioned above and compared with the theoretical curve. *At a glance*, from Fig. 1 it is possible to see the detrimental effect caused by a relatively low DIP value on all three interpolation processes, especially at large lag-times, where the Spline function performs worse. In order to quantify such a discrepancy, we have evaluated the relative absolute error (RAE(*t*)) of the three interpolation functions ($g_{I}(t)$) with respect to the theoretical functions computed by means of Eqs. (2) and (3):

7 $$\begin{aligned} RAE(t)=\frac{|g_{I}(t)-A(t)|}{A(t)}~~~or~~~RAE(t)=\frac{|g_{I}(t)-\Pi (t)|}{\Pi (t)} \end{aligned}$$

The insets in Fig. 1 show the RAE(*t*) of the three interpolation functions, with a focus in the time window ranging from $10^1$s to $10^2$s, where the error is at its highest. Interestingly, in the case of the single exponential decay, all three RAEs increase rapidly by almost ten decades across the explored time window, with the Spline function returning the highest error. A similar outcome can be seen in the case of $\Pi (t)$, where both PCHIP and Makima interpolation functions perform significantly better than the Spline function, returning a RAE(*t*) at long times almost five decades smaller. Notice that, the minima in the RAE(*t*) are due to the inherent nature of interpolation functions to pass through each experimental data point; a condition that is not guaranteed by any fitting procedure.

**Figure 2:**
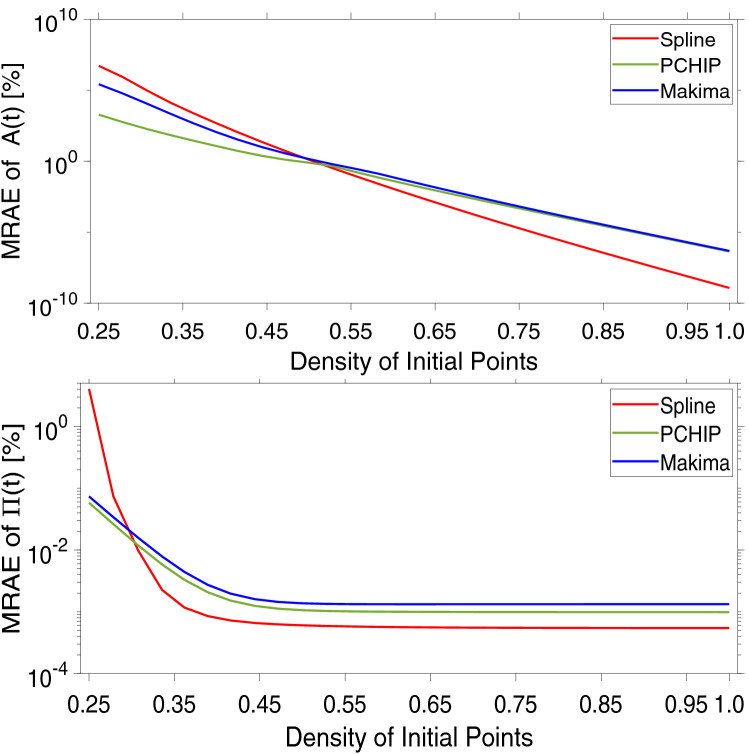
Mean relative absolute error (MRAE) vs. the density of initial experimental points (DIP) of the three MATLAB built-in interpolation functions: Spline, PCHIP and Makima. (Top) The MRAE is evaluated with respect to Eq. (2). (Bottom) The MRAE is evaluated with respect to Eq. (3).

In order to investigate the fidelity of the interpolation process as function of DIP, we have evaluated the mean relative absolute error (MRAE) of the interpolating functions for $N_e$ varying from 10 to 10^4^, which implies a DIP ranging from 1/4 to 1 (see Fig. 2). The MRAE is defined as follows:

8 $$\begin{aligned} MRAE=\frac{100}{N} \sum _{n=1}^{N} \frac{|g_{I}(n)-A(n)|}{A(n)} \end{aligned}$$

where *N* is the number of points at which all the functions are evaluated within the experimental time window $[10^{-2},10^2]$; in this study $N=10^4$. Notice that, a similar expression to Eq. (8) can be written in terms of $\Pi (t)$. From Fig. 2 it is interesting to note that, at relatively low DIP values, all three functions perform poorly; with the Spline function performing worst and the PCHIP returning the lowest error in both the cases; yet higher than $10^3\%$ in the case of the single exponential decay (Fig. 2, top). We argue that, the better fidelity shown by the PCHIP function can be attributed to the non continuous nature of the second-derivative of its interpolation algorithm, which prevents from overshoots and returns much lower oscillation in case of large jumps between data points (i.e., at low DIP) when compared to the Spline function.

Notably, for DIP values approaching 1, all three interpolation functions successfully recover the sampled functions to a high degree of accuracy, returning MRAE values lower than 10^-^^5^% for the case of the single exponential decay. In this case, from a practical point of view, it is worth noting that the MRAE of all three interpolation functions falls below 1% for DIP values higher than circa 0.56, which implies a minimum number of 174 initial sampled points within the explored time window. Interestingly, when this outcome is applied for instance to DLS measurements (for which the experimental time window spans from 10^−7^ sec to 10^2^ sec), a DIP of 0.56 would imply a minimum number of $\approx 10^5$ initial timestamps (or lag-times); a condition never met in real experiments, where instead a DIP of circa 0.25 is commonly found. In the case of the NMSD (see Fig. 2, bottom), at low DIP values, both PCHIP and Makima start with a MRAE smaller than 0.1%, while the Spline function starts with a MRAE value of circa 4%; thus confirming its poor performance at relatively low DIP values. Nonetheless, when comparing the errors generated by the interpolation procedures for the two cases of study, it is striking the different behaviour of their MRAE curves, which differ from each other by almost five orders of magnitude in opposite direction at the extremes of the explored range of DIP values.

**Figure 3:**
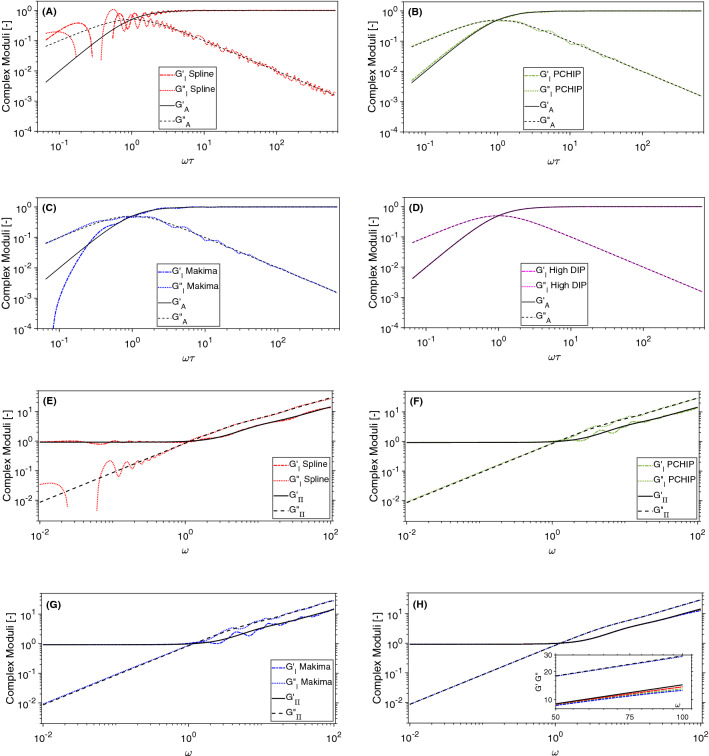
Comparison between the frequency-dependent complex moduli obtained via Eq. (4) and those evaluated by Fourier transforming via Eq. (1) the interpolations functions shown in Fig. 1 for DIP $=1/4$ in (**A**, **B**, **C**, **E**, **F**, **G**) and for DIP $=1$ in (**D**, **H**).

Let us now investigate how the error propagates into the frequency-domain as function of both the chosen interpolation algorithm and the DIP, when performing the Fourier transform by means of the Evans & Tassieri’s method. In Fig. 3, the viscoelastic moduli evaluated by means of Eqs. (4) and (5) are drawn together with those derived by Fourier transforming the interpolation functions shown in Fig. 1 for the case of DIP $=1/4$.

From Fig. 3, it is evident the detrimental effects of using a relatively low DIP value for determining the dynamic information embedded within the sampled functions. In particular, as discussed earlier, the Spline function carries the biggest error because of its poor performance in resembling the sampled functions at long times, which translates into artefacts in the low-frequency behaviour of both the moduli. Notably, these artefacts are significantly reduced in the case of Makima and even further in the case of PCHIP, which is the one that performs best at low DIP values. In Fig. 3 we also report the viscoelastic moduli evaluated in the case of DIP $=1$ as a means of comparison. The inset of Fig. 3h highlights the relatively small divergence of the elastic modulus from the expected value at high frequencies shown by all three of the interpolation functions. We argue that this (small) discrepancy is caused by the different constrains on the first and second derivatives adopted by the three interpolation algorithms to model the sampled function at the boundaries of the experimental time window; i.e, here within the time-gap occurring between the first experimental point at $t_1$ and the asymptotic one at $t=0$.

**Figure 4:**
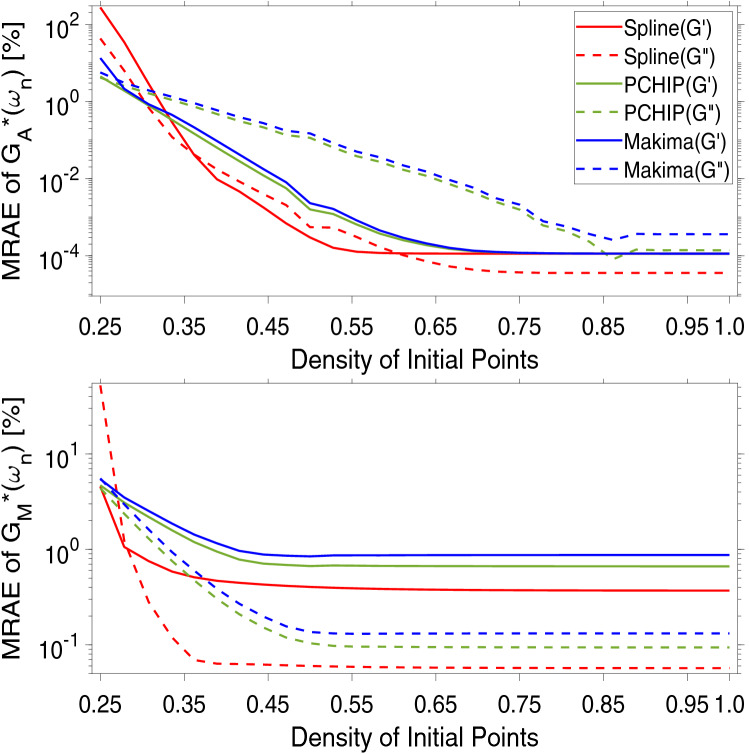
Mean relative absolute error (MRAE) of the frequency-dependent complex moduli determined by Fourier transforming (via Eq. 1) the interpolation functions shown in Fig. 1 (top & bottom) for DIP values ranging from 1/4 to 1.

In order to quantify these discrepancies, we have evaluated the MRAE of both the real and imaginary parts (i.e., $G'_{I}(\omega )$ and $G''_{I}(\omega )$) of the calculated complex modulus with respect to their expected values (i.e., $G'_{A}(\omega )$ and $G''_{A}(\omega )$, $G'_\Pi(\omega )$ and $G''_\Pi(\omega )$) obtained from Eqs. (4) and (5), respectively:

9 $$\begin{aligned} MRAE =\frac{1}{N_\omega }\sum _{n=1}^{N_\omega }\frac{|G'_{I}(\omega _n)-G'_{A}(\omega _n)|}{G'_{A}(\omega _n)} \end{aligned}$$

where $n=1...N_\omega$ is the number of frequencies at which Eq. (9) is evaluated (here $N_\omega =200$, with $\omega _n$ equally spaced on a logarithmic scale ranging from $10^{-2}$ to $10^2$ Hz). A similar expression can be written for the viscous modulus, with $G'_{I}(\omega _n)$ and $G'_{A}(\omega _n)$ replaced by $G''_{I}(\omega _n)$ and $G''_{A}(\omega _n)$, and for Eq. (5) by replacing $G^*_{A}(\omega _n)$ with $G^*_\Pi(\omega _n)$, respectively. In particular, we have evaluated the MRAE for DIP values ranging from 1/4 to 1; and the results are reported in Fig. 4 for both the sampled functions.

From Fig. 4 top, it can be seen that (i) at relatively low DIP values (i.e., for DIP$\lesssim 0.33$) both the interpolation functions PCHIP and Makima perform significantly better than the Spline one; which, (ii) for DIP $>0.33$ returns relatively lower values of the MRAE for both the moduli. Interestingly, for DIP $>0.9$ the MRAE reaches a plateau value for both the moduli and all the interpolation functions; with the MRAEs of the elastic modulus showing an identical value for all three interpolation functions starting from DIP $=0.75$. Moreover, it can be seen that for $0.33<$DIP $<0.9$ both PCHIP and Makima return a MRAE of the viscous modulus much higher than for the elastic one, which is actually comparable to the MRAE of both the moduli obtained by means of a Spline interpolation. From Fig. 4 top, we can assert that, all the three interpolation functions provide a MRAE of both the moduli smaller than 1% for DIP $>0.4$, which implies the need of a minimum number of initial data points of circa 40 within the explored time window.

From Fig. 4 bottom, it can be seen that (i) at relatively low DIP values (i.e., for DIP $\lesssim 0.28$) both the interpolation functions PCHIP and Makima return a similar value of the MRAE (i.e. lower than $5\%$) for both the moduli; whereas, the Spline function returns a similar value of the MRAE only for the elastic modulus, while the MRAE for the viscous modulus goes up to a value of $50\%$ at DIP $=0.25$. Interestingly, for DIP $>0.5$ the MRAE of both the viscoelastic moduli reach similar constant values for all the interpolation functions. Specifically, the MRAEs of the elastic modulus is of the order of $1\%$ and the MRAEs of the viscous modulus is of the order of $0.1\%$. Notably, also in this case, the Spline function returns overall a lower (and constant) MRAE than both PCHIP and Makima for DIP $>0.4$.

Let us now investigate how the presence of (white) noise impacts on the effectiveness of the analytical method developed by Evans & Tassieri, when the above mentioned interpolation functions are employed to analyse both Eqs. (2) and (3), sampled at a fixed acquisition rate and for which the amplitude of the added noise is varied to explore a range of signal-to-noise ratios (SNR) spanning from 1dB to 350dB. The latter has been calculated by using the following equation:

10 $$\begin{aligned} SNR=\Bigg (\frac{A_{signal}}{A_{noise}}\Bigg )^2, \end{aligned}$$

where $A_{signal}$ and $A_{noise}$ are the root mean square amplitudes of the signal and the noise, respectively.

**Figure 5:**
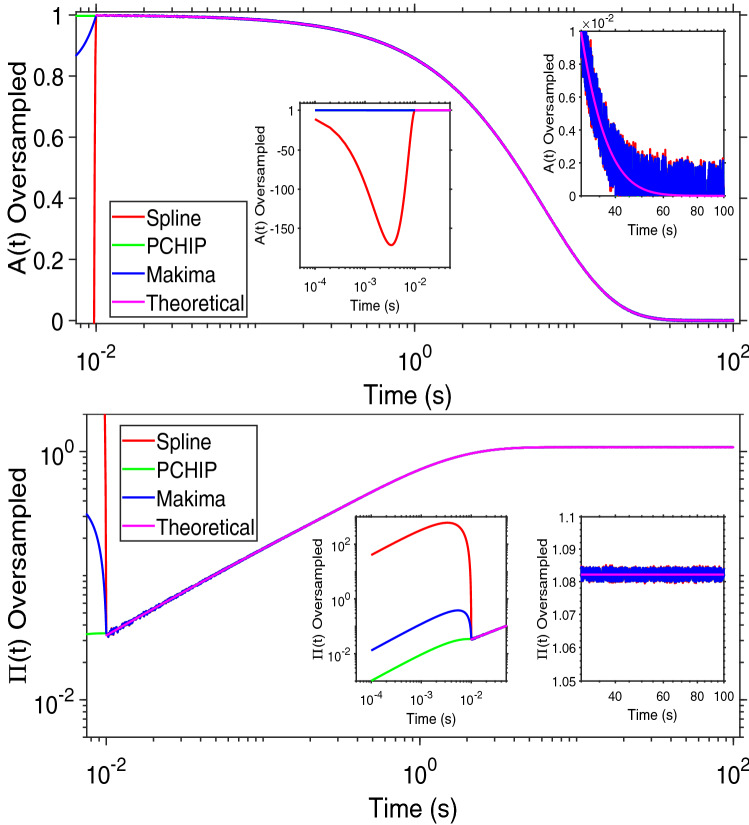
(Top) Eq. (2) and (bottom) Eq. (3) drawn as continuous (pink) lines by using $10^4$ experimental points linearly spaced in time. A white noise having a SNR $=50$ is added to the experimental data, which are then interpolated by means of three MATLAB built-in interpolation functions: Spline, PCHIP and Makima. The insets highlight the detrimental effects on the interpolation process due to the presence of noise, both at short and long time scales.

As a means of discussion, in Fig. 5 are reported both Eqs. (2) (top) and (3) (bottom) drawn as continuous (pink) lines by using $10^4$ experimental points linearly spaced within a time window of $[10^{-2},10^2]$ sec (i.e., DIP $=1$), to which a random white noise having a SNR $=50$ has been added. The resulting ‘noisy’ functions have been then interpolated by means of all three of the above mentioned MATLAB built-in interpolation functions. From Fig. 5, and further elucidated hereafter, it can be seen that, at relatively low SNR, the different nature of the interpolation functions can lead to very large deviations from the expected values within the time window $[0,t_1]$, where $t_1=1/AR$. This is especially true in the case of the Spline function, as shown by the insets of Fig. 5, both at top and bottom.

**Figure 6:**
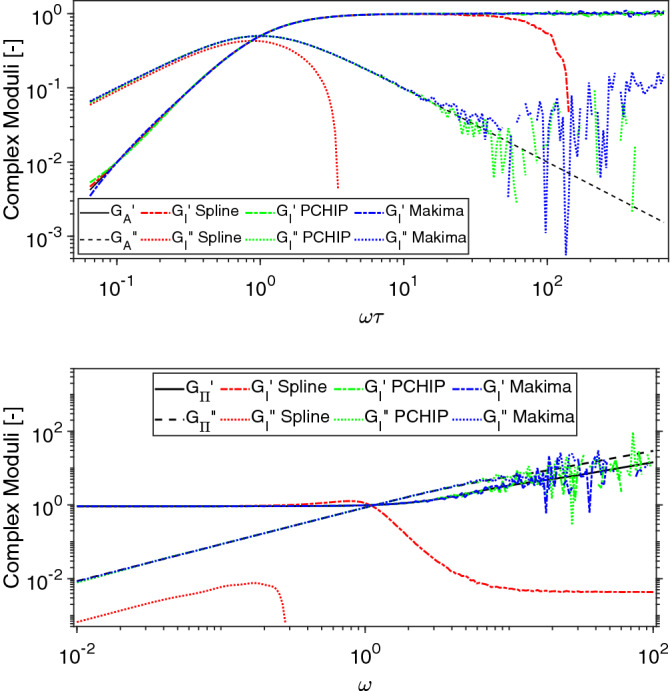
Comparison between the frequency-dependent complex moduli obtained via Eqs. (4) (top) and (5) (bottom) and those evaluated by Fourier transforming via Eq. (1) the interpolations functions shown in Fig. 5.

**Figure 7:**
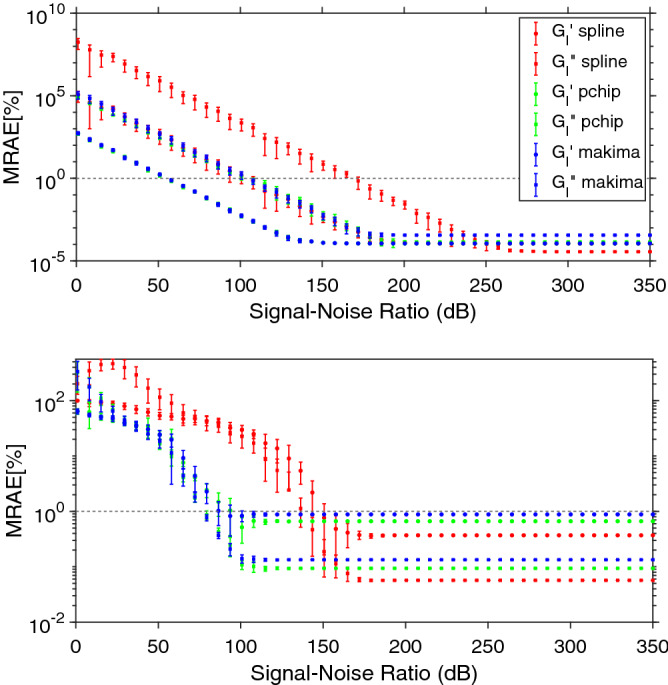
Mean relative absolute error (MRAE) of the frequency-dependent complex moduli determined by Fourier transforming (via Eq. 1) the interpolation functions shown in Fig. 5 (top & bottom respectively) for SNR values ranging from 1 to 350 dB. The error bars represent one standard deviation of uncertainty calculated over ten repeats.

In contrast, due to its non-continuous nature of the second derivative that prevents overshoots, the PCHIP algorithm performs better than both Spline and Makima functions. Notably, as we shall demonstrate below, these deviations are the major source of error at high frequencies when performing the Fourier transform via Eq. (1), which otherwise reveals to be almost unaffected at low frequencies by the presence of the noise themselves; as shown in Fig. 6, but also supported by the experimental evidences reported in Figures 7 and 8 of Ref.^14^. Here, Fig. 6 shows a comparison between the viscoelastic moduli calculated via Eqs. (4) (top) and (5) (bottom) and those evaluated by Fourier transforming via Eq. (1) the interpolations functions shown in Fig. 5. From Fig. 6 it is clear that the high frequency noise caused by the ‘miss modelling’ of the short time (i.e., $\forall t\in ]0,t_1[$) behaviour of the experimental data tends to spill over into the top of the experimental frequency range, with the moduli evaluated either via PCHIP or Makima adhering most to the exact solutions, especially at relatively low frequencies; whereas the Spline function performs worse over the whole frequency range. Interestingly, also in this case, for a given set of experimental data, there exists a threshold value of the SNR above which all the three interpolation functions allow an accurate estimation of the Fourier transform.

In order to identify the threshold value for each interpolation method applied to both Eqs. (2) and (3) (with DIP $=1$), we have evaluated the MRAE of the transformed data (i.e., Eqs. 9) as function of the SNR; with the latter ranging from 1dB to 350 dB. The results are reported in Fig. 7 for both the sampled functions shown in Fig. 5. From Fig. 7 top, it can be seen that at low values of SNR (i.e. $\lesssim 50$ dB) all of the interpolation functions perform poorly, with a MRAE as large as $10^8\%$ for the Spline interpolation function at SNR $=0$; thus performing significantly worse than PCHIP and Makima. However, as one would expect, by increasing the SNR to a relatively high value (here above $\sim 170$dB) the MRAEs of the moduli associated with each interpolation function fall below $1\%$ and asymptotically approach the values presented in Fig. 4 for DIP $=1$. Interestingly, from Fig. 7, one could argue that, in presence of noise, the interpolation processes performed by PCHIP and Makima work best, when compared to the Spline. However, in response it must be highlighted that, in real experiments, it is rare to process data with low SNR; such as those reported in the insets of Fig. 5, where both the Eqs. (2) and (3) are drawn with a SNR $=50$, which would commonly be discarded as ‘noisy measurements’.

## Conclusion

In this article we have presented an open-access code named i-RheoFT that allows to evaluate the Fourier transform of any generic time-dependent function that vanishes for negative times, sampled at a finite set of data points that extend over a finite range, and *need not* be equally spaced. The analytical method that underpins this code has been originally introduced by Evans & Tassieri^17,19^ in the form of an open-access LabVIEW executable specialised for the analysis of microrheology measurements performed with optical tweezers. Here we expand the range of its potential applications by implementing it into an open-access MATLAB code, with the aim of reaching a broader audience and encouraging its exploitation in a variety of applications.

The effectiveness of i-RheoFT has been corroborated here by evaluating the Fourier transform of two generic functions having a known analytical expression of their Fourier transforms. Moreover, the analytical method has been tested as function of three important experimental parameters: (i) the ‘density of initial experimental points’ (DIP) describing the sampled function; (ii) the interpolation algorithm used to perform the “*virtual oversampling*” procedure introduced by Evans & Tassieri, which here is achieved by means of the following three built-in MATLAB functions: Spline, Makima and PCHIP; and (iii) the destructive effects on the expected outcomes due to the presence of (white) noise.

The outcomes of this study reveal that, at relatively high DIP values and signal-to-noise ratios, all three interpolation functions perform well in recovering the original information of the sampled function, with the Spline function always performing best. Whereas, by reducing *either* the number of initial data points *or* the signal-to-noise ratio, there exists a threshold value below which all three functions perform poorly, with the Spline function always returning the highest error of all three functions.

Therefore, we envisage that these results and i-RheoFT will be of particular interest and use to all those (experimental and simulation) studies where fast streams of acquired data are processed on-the-fly to build time-averaged functions, which are often defined by a finite number of data points over a limited time window spanning several decades; as for instance, in the cases of diffusing wave spectroscopy and dynamic light scattering measurements.

## Supplementary Information

Supplementary Information.
