# Supplementary material for: i-RheoFT: Fourier transforming sampled functions without artefacts
Source: Sci Rep. 2021 Dec 15;11:24047. doi: 10.1038/s41598-021-02922-8 (PMC8674267; doi:10.1038/s41598-021-02922-8)
Supplement: Supplementary file 1 — Supplementary Information. [file 41598_2021_2922_MOESM1_ESM.pdf]

Supplementary Info "*i-RheoFT: Fourier transforming sampled functions without artefacts*"

Matthew G. Smith,[1] Graham M. Gibson,[2] and Manlio Tassieri[1,*]

[1]*Division of Biomedical Engineering, James Watt School of Engineering, University of Glasgow, Glasgow, G12 8LT, UK*

[2]*SUPA, School of Physics and Astronomy, University of Glasgow, Glasgow, G12 8QQ, UK*

(Dated: August 11, 2021)

I. MATLAB SCRIPT

In this section we report the MATLAB script that has been used in this work to evaluate the Fourier transform of two generic functions defined by Equations 2 and 3 in the main text, which are evaluated for a finite set of timestamps. A "readme" file is reported in the next section.

```
%% I-RheoFT: Fourier transforming sampled functions without artefacts.

% Authors: Matthew G. Smith, Graham M. Gibson & Manlio Tassieri
% University of Glasgow
% Date: 14/09/2020

% Aim: This program is designed to evaluate the Fourier transform (FT)
% of a generic function, sampled at a finite rate. It will produce the FT
% named GFT and the Complex modulus in the form G' and G" named gdata_I.
% When the% script is run it will ask for the file name (.csv/.xlsx),
% the value of the function at time = 0 (g0), the gradient of the function
%  at time=+infinity (ginf) and the interpolation function you wish to use
% (spline, makima or pchip). As recommended in the paper, if the function
% has a Density of Initial Points (DIP) of more than 0.35 then the spline
% function should be chosen as this gives the lowest error.
% The oversampling (OverSample) and the number of frequency points
% (freqpoints) can be edited, although a significant increase in either
% could lead to longer run times.

%% Ask user for File, g0, g_infinity and interpolation function

filename = input('Please input file name: ','s');
file = readtable(filename);
g0 = input('Please input g0: ');
```

```
ginf = input('Please input ginf: ');
InterpFunc = ...
    input('Please input the Interpolation Function(spline,makima,pchip): ','s');

file = table2array(file);
t = file(:,1).';
g = file(:,2).';
t0 = 0;
Num_Init_Pts = length(t);
freqpoints = 200;
OverSample = 1e5*Num_Init_Pts;

%% Interpolation, Oversampling and Fourier transform

% Interpolation and Oversampling
t_I = linspace(t(1),t(end),OverSample);
Gint_I = interp1(t,g,t_I,InterpFunc);

% Fourier Transform using function below
[GFT,gdata_I] = IRHEO_GT(t_I,Gint_I,freqpoints,g0,ginf);



%% The Fourier Transform function
function [GFT,gdata_I] = IRHEO_GT(t_I,Gint_I,freqpoints,g0,ginf)

gdata_I = zeros(freqpoints,3);
GFT = zeros(freqpoints,3);
wrange = logspace(-2,2,freqpoints); % Freqency Range

A = zeros(1,length(t_I)-1);
```

```
for n = 1:freqpoints
        w = wrange(n);
    for k = 2:length(t_I)
        A(k) = (((Gint_I(k)-Gint_I(k-1))/(t_I(k)-t_I(k-1)))*...
            (exp(-i*w*t_I(k-1))-exp(-i*w*t_I(k))));
    end
    GFT1 = ((i*w*g0+((1-exp(-i*w*t_I(2)))*((Gint_I(1)-g0)/t_I(2)))+...
        ginf*exp(-i*w*t_I(end)))+sum(A))/(i*w)^2;
    Gstar = GFT1*(i*w);
    GFT(n,:) = [w real(GFT1) imag(GFT1)];
    gdata_I(n,:) = [w real(Gstar) imag(Gstar)];
end
end
```

II. README

This is a MATLAB code that implements the analytical method originally introduced by Evans & Tassieri[1,2], which allows to evaluate the Fourier transform of any generic time-dependent function that vanishes for negative times, sampled at a finite set of data points that extend over a finite range, and need not be equally spaced.

In particular this program has been designed to evaluate the Fourier transform of data fed in the form of a .csv/.xlsx file, while also giving the user the option to use any of the three interpolations functions described in the main manuscript.

A. Installation

The .m file to be installed is *iRheoFT.m*, which is written in MATLAB R2018b. It is recommended that the input data should be in a column format in a .csv or .xlsx file. Attention must be paid to make sure that both the data and the .m file are in the same directory in MATLAB; otherwise the data file will not be found.

B. How to use?

Once the files have correctly been installed in the same directory of MATLAB, running the script will produce a series of prompts for the user to input. Firstly, it will ask the user to input the file name including the file type (.csv/.xlsx). It will then ask the user to input the value of the function at time 0 and then the value of the gradient of the function at time plus infinity. Finally, it will ask the user to input which interpolation method they would like to use (Spline, Makima or PCHIP) and this should be chosen depending on the Density of Initial Points (DIP) of the data analysed. The DIP can be calculated from Equation 6. One can also edit the Oversampling and the number of frequencies used, although the default values should be valid in most of the cases.

Once the script has finished running, the Fourier transform of the function will be contained in the variable GFT and the Complex Modulus will be contained in the variable $gdata\_I$.

C. Example Data

A possible starting point to make sure the program is working properly, would be to generate a single exponential decay function that takes the form of Equation 2. The relaxation time used in the manuscript is 6.5 sec, with 10^3 points logarithmically spaced in a time window ranging from $0.01 - 100$ sec. The data should be saved as a .csv or a .xlsx file and loaded into the same directory of *iRheoFT.m*. In order to produce the viscoelastic moduli curves, one should plot the log-log of $gdata\_I$, where column 1 is the frequency, column 2 is G' and column 3 is G''. The plot should be similar to Figure 3 (D), if this is the case then the code is working as intended.

---

* Corresponding author: Manlio.Tassieri@glasgow.ac.uk

[1] R. M. L. Evans, Manlio Tassieri, Dietmar Auhl, and Thomas A. Waigh. Direct conversion of rheological compliance measurements into storage and loss moduli. *Physical Review E*, 80(1), July 2009.

[2] Manlio Tassieri, R M L Evans, Rebecca L Warren, Nicholas J Bailey, and Jonathan M Cooper. Microrheology with optical tweezers: data analysis. *New Journal of Physics*, 14(11):115032, nov 2012.
